# Special Issue “Molecular Insight into Alzheimer’s Disease”

**DOI:** 10.3390/ijms27146385

**Published:** 2026-07-18

**Authors:** Catherine Malaplate, Thierry Oster

**Affiliations:** 1 Nutrition, Génétique, Exposition aux Risques Environnementaux (NGERE), Inserm U1256, Campus Brabois, University of Lorraine, 54500 Vandœuvre-lès-Nancy, France; 2 Department of Biochemistry, Molecular Biology and Nutrition, Nancy University Hospital, 54000 Nancy, France

## 1. Introduction

Alzheimer’s disease (AD) remains one of the greatest biomedical challenges of our time, mostly because it appears to result from a complex interplay of several determinants. Despite decades of intensive investigation, no single hypothesis has fully explained the complexity of disease onset and progression, nor had any therapeutic strategy succeeded in consistently halting neurodegeneration thus far.

The title of this Special Issue, Molecular Insights into Alzheimer’s Disease, reflects a central ambition of contemporary AD research: to decipher the molecular mechanisms that underlie disease initiation and progression. Far from challenging the importance of amyloid and tau, these molecular insights presented in this Special Issue illustrate an critical conceptual evolution: rather than focusing on a single pathogenic cascade or isolated pathogenic mechanisms, they increasingly reveal biological networks through which these hallmark pathologies interact with vascular dysfunction, lipid homeostasis, oxidative stress, regenerative capacity, and genetic susceptibility to shape disease expression [1]. This evolving perspective is reshaping both our understanding of disease mechanisms and the strategies proposed for diagnosis, prevention, and treatment.

Although the contributions assembled in this Special Issue address diverse scientific questions using complementary experimental and computational approaches, they converge toward a common message: progress in AD research increasingly depends on integrating multiple complementary dimensions of disease biology rather than seeking a single causative pathway. The conceptual framework proposed by Scarano et al. provides a valuable lens through which these contributions can be interpreted. While their review advocates moving beyond reductionist models toward a multifactorial understanding of AD, the accompanying articles offer concrete illustrations of this important transition.

Several articles revisit molecular mechanisms that have received comparatively less attention than amyloid and tau while demonstrating their potential relevance to disease pathogenesis. Volynsky et al. demonstrate how membrane sterol–APP interactions influence amyloid precursor protein processing, suggesting that membrane organization and the lipid environment itself may represent a modifiable determinant of amyloidogenesis. Likewise, Mironenko et al. show that functional characterization of rare angiotensin I-converting enzyme (ACE) variants may reveal previously unrecognized determinants of individual susceptibility, illustrating the growing importance of functional phenotyping beyond conventional genetic analyses. Complementing these findings, Khan highlights the intricate relationships linking iron homeostasis, oxidative stress, and APP expression, while Kim & Moon provide a comprehensive overview of NADPH oxidase isoforms as important contributors to oxidative stress and neuroinflammation. Together, these studies portray AD as the consequence of interacting molecular systems rather than isolated pathogenic cascades.

Another emerging theme concerns the growing recognition that neuronal dysfunction cannot be fully understood without considering its surrounding biological environment. Joseph et al. demonstrate that cerebral small vessel disease and AD exhibit distinct perfusion signatures despite a shared blood–brain barrier dysfunction, reinforcing the importance of neurovascular integrity in cognitive decline rather than secondary consequences of neurodegeneration. At the same time, the review by Dwamena et al. emphasizes that the adult hippocampus retains endogenous regenerative potential, opening perspectives that extend beyond preventing neuronal loss toward promoting neuronal replacement and functional recovery. Collectively, these contributions expand the biological landscape of AD by emphasizing the critical roles of the neurovascular unit, membrane dynamics, circulating biomarkers, and regenerative mechanisms.

A second major convergence emerging from this Special Issue is the movement toward precision medicine. AD is increasingly viewed not as a single disease entity but as a heterogeneous spectrum of biological subtypes that may require individualized diagnostic and therapeutic strategies. This evolution is particularly illustrated by Mironenko et al. who demonstrate how blood-based functional phenotyping of ACE variants may complement genomic information to improve individual risk stratification. Similarly, Tandon et al. apply data-driven neuroanatomical staging using structural MRI and machine-learning approaches to identify distinct patterns of disease progression beyond traditional clinical classifications. Together, these studies exemplify a broader shift from symptom-based diagnosis toward biologically informed patient characterization.

Equally striking is the remarkable methodological diversity represented across these contributions. Advanced neuroimaging, arterial spin labeling, structural biology, nuclear magnetic resonance spectroscopy, molecular dynamics simulations, computational disease modeling, biomarker phenotyping, and integrative reviews collectively illustrate how technological innovation continues to transform AD research. Rather than simply generating larger datasets, these complementary methodologies allow increasingly sophisticated interrogation of disease mechanisms across multiple spatial and temporal scales.

Beyond their individual findings, the contributions published in this Special Issue by Scarano et al., Volynsky et al., Khan, Kim et al., Mironenko et al., Joseph et al., Dwamena et al., and Tandon et al. collectively illustrate an important conceptual evolution. Rather than viewing AD through the lens of isolated pathological hallmarks, they advocate a more integrated understanding in which genetic susceptibility, vascular dysfunction, lipid biology, oxidative stress, impaired regenerative capacity, and environmental influences interact within dynamic biological networks. This systems-level perspective does not diminish the importance of amyloid or tau; instead, it situates these hallmarks within a broader biological framework that better reflects the marked heterogeneity observed between patients and may ultimately facilitate the development of more personalized interventions.

## 2. Highlights of the Contributions

### 2.1. Part I: Rethinking Alzheimer’s Disease

#### Alzheimer’s Disease Aetiology Hypotheses and Therapeutic Strategies: A Perspective

Scarano et al. reviewed current hypotheses on AD etiology and critically examined therapeutic strategies spanning neurotransmitter and ion modulation, amyloid and tau targeting, and broader disease-modifying approaches. Synthesizing evidence from experimental and clinical research, they discussed genetic susceptibility, environmental risk factors, and neuroprotective pathways while emphasizing the limitations of currently approved treatments, suggesting synergistic effects from concomitant key factors. The authors advocate a multidimensional view of AD integrating genetic, molecular, and environmental determinants. Their analysis supports the development of personalized, multi-target therapeutic strategies better suited to the complex and heterogeneous biology of AD.

### 2.2. Part II: Molecular Determinants

#### 2.2.1. Diverse Interactions of Sterols with Amyloid Precursor Protein Transmembrane Domain

Volynsky et al. investigated how sterols regulate amyloid precursor protein (APP) processing through interactions with its transmembrane domain, considering the influence of different lipid species on these interactions [2]. Combining solution and solid-state NMR spectroscopy with molecular dynamics simulations in distinct lipid environments, they identified multiple sterol-binding sites capable of shifting APP toward alternative amyloidogenic or non-amyloidogenic processing pathways. Their findings extend previous work on cholesterol-dependent APP regulation by demonstrating that local membrane composition critically modulates these interactions. Rather than acting solely through cholesterol abundance, membrane lipid organization emerges as a key determinant of Aβ production. This work highlights membrane biophysics as a promising avenue for developing therapeutic strategies that selectively modulate APP processing without directly targeting secretase activity.

#### 2.2.2. Iron-Mediated Overexpression of Amyloid Precursor Protein via Iron Responsive mRNA in Alzheimer’s Disease

Khan examined how iron dysregulation may promote AD through iron-responsive regulation of amyloid precursor protein (APP) expression. Drawing on molecular, biochemical, and translational evidence, the review describes how excess iron enhances APP translation via iron-responsive mRNA elements, thereby increasing amyloidogenic processing and oxidative stress. Building upon the foundational work on APP iron-responsive elements and subsequent studies linking iron accumulation to neurodegeneration [3], the author proposes iron homeostasis as a central driver of Alzheimer’s pathology. These findings reinforce the rationale for developing therapeutic strategies targeting iron metabolism and ferroptosis-related pathways alongside conventional anti-amyloid interventions in AD.

#### 2.2.3. Molecular Roles of NADPH Oxidase-Mediated Oxidative Stress in Alzheimer’s Disease

Kim et al. reviewed the isoform-specific contributions of NADPH oxidase (NOX) enzymes to oxidative stress and neuroinflammation in AD. Integrating molecular, cellular, and preclinical evidence, they examined how distinct NOX isoforms contribute to reactive oxygen species production, amyloid pathology, tau hyperphosphorylation, synaptic dysfunction, and neuronal loss. Building on pioneering studies implicating oxidative stress in AD [4], the authors highlight the differential roles of individual NOX isoforms and discuss emerging isoform-selective inhibitors as therapeutic candidates. Their review suggests that targeting specific NOX enzymes may provide a more precise approach to limiting oxidative damage while preserving physiological redox signaling in AD.

#### 2.2.4. ACE-Dependent Alzheimer’s Disease

Mironenko et al. investigated whether rare heterozygous angiotensin 1-converting enzyme (ACE) variants associated with reduced circulating angiotensin-converting enzyme levels increase susceptibility to late-onset AD as previously hypothesized [5]. Using blood samples from 147 carriers of 23 rare ACE variants and 70 controls, they combined ACE activity assays, monoclonal antibody-based conformational fingerprinting, and genotype–phenotype analyses. Several variants, including the relatively frequent Y215C mutation, were associated with markedly reduced circulating ACE levels and a trend toward increased dementia, hippocampal dysfunction, and frontal impairment. Building on previous ACE phenotyping studies, they identified blood-based phenotypic markers capable of recognizing pathogenic ACE variants. These findings support functional ACE phenotyping as a promising biomarker strategy for early risk stratification and precision medicine in AD, although larger longitudinal studies are needed to establish its clinical utility.

### 2.3. Part III: Beyond Neurons

#### 2.3.1. Dementia from Small Vessel Disease Versus Alzheimer’s Disease

Joseph et al. investigated whether cerebral small vessel disease and Alzheimer’s disease represent distinct or overlapping vascular pathologies. Using 3D arterial spin labeling MRI, FLAIR, susceptibility-weighted imaging, cognitive assessment, and APOE genotyping in a pilot cohort, they identified distinct arterial and capillary perfusion signatures despite shared blood–brain barrier dysfunction. Interpreting their findings in light of seminal work on cerebral small vessel disease [6] and on blood–brain barrier dysfunction [7], the authors suggest that advanced perfusion imaging could improve differential diagnosis and support tailored therapeutic strategies targeting microvascular dysfunction. More broadly, the study reinforces the growing recognition of vascular dysfunction as a key therapeutic avenue in AD.

#### 2.3.2. Boosting Neurogenesis as a Strategy in Treating Alzheimer’s Disease

Dwamena et al. reviewed the molecular mechanisms governing adult hippocampal neurogenesis and their dysregulation in Alzheimer’s disease. Integrating evidence from experimental and clinical studies, they examined transcriptional, epigenetic, inflammatory, and metabolic pathways controlling neural stem cell function, together with emerging therapeutic approaches including pharmacological agents, stem cell therapy, gene therapy, and epigenetic modulation. Building upon landmark discoveries demonstrating persistent human neurogenesis in aged adults and AD patients [8], the authors argue that restoring endogenous neuronal regeneration may complement conventional anti-amyloid and anti-tau strategies. Their review positions neurogenesis as a promising regenerative target that could enhance cognitive resilience and support disease-modifying therapies for AD.

### 2.4. Part IV: Toward Precision Medicine

#### Comprehensive Anatomical Staging Predicts Clinical Progression in Mild Cognitive Impairment

Tandon et al. developed a data-driven neuroanatomical staging framework to predict progression from mild cognitive impairment to AD. Using structural MRI data from the Alzheimer’s Disease Neuroimaging Initiative (ADNI) and the scalable Subtype and Stage Inference (s-SuStaIn) framework [9], they identified four distinct anatomical subtypes characterized by different progression trajectories, biomarker profiles, and cognitive decline patterns. The model outperformed conventional demographic and genetic predictors of conversion from MCI to AD while capturing clinically meaningful disease heterogeneity. These findings support MRI-based anatomical staging as a valuable tool for personalized prognosis, patient stratification, and optimization of clinical trial design, illustrating the shift toward precision medicine in AD.

## 3. Conclusions

The contributions assembled in this Special Issue echo the growing consensus that future advances in AD research will rely on integrating diverse molecular, cellular, vascular, regenerative, imaging, and computational perspectives into a coherent biological framework [10]. As Guest Editors, we hope that this collection stimulates further interdisciplinary collaborations and encourages investigators to continue bridging molecular biology, neuroimaging, vascular neuroscience, computational modeling, and translational medicine and research toward a more systems-level understanding of AD. This evolution is consistent with recent calls to move beyond reductionist models and build multidimensional frameworks capable of guiding future diagnostic and therapeutic innovations. The future of AD research will likely depend not on identifying a single dominant mechanism, but on understanding how multiple interconnected processes collectively shape AD onset and progression.
